# The role of family environment and parental factors: a person-oriented study of adolescents’ psychological distress and help-seeking patterns

**DOI:** 10.1186/s13034-025-00986-2

**Published:** 2025-11-24

**Authors:** Na Lyu, Qing-Yao Xue, Xin Li, Shu Yan, Mo Chen, Hao Hou, Dan Luo, Chen Qian, Pei Zhang, Yang Zhou, Bing Xiang Yang

**Affiliations:** 1 Department of Psychiatry, Wuhan Mental Health Center, No. 89, Gongnongbing Road, Jiang’an District, Wuhan, 430012 Hubei Province China; 2 Department of Psychiatry, Wuhan Hospital for Psychotherapy, Wuhan, 430012 Hubei Province China; 3 Department of Psychology, University of Alberta, Edmonton, AB Canada; 4 Center for Wise Information Technology of Mental Health Nursing Research, School of Nursing, Wuhan University, Wuhan, 430071 Hubei Province China; 5 Department of Psychiatry, Wuhan Children’s Hospital of Tongji Medical College, Huazhong University of Science and Technology, Wuhan, 430012 Hubei Province China

**Keywords:** Family environment, Parental factors, Adolescents, Latent profile analysis, Distress, Help-seeking

## Abstract

**Background:**

Adolescents’ mental health is shaped by their coping strategies and the broader family context in which they live. However, few studies have examined psychological distress and help-seeking patterns jointly, especially from a person-oriented perspective. Understanding distinct adolescent risk profiles and how family and parental factors influence them may inform more effective prevention strategies. This study aimed to: (1) identify latent profiles of adolescents based on their psychological distress and help-seeking intentions; and (2) explore how family and parental factors predict profile membership and self-harm risk.

**Methods:**

A cross-sectional study was conducted in 2021 with 7,934 Chinese secondary school students and one parent per adolescent. Adolescents completed validated measures of depression, anxiety, and help-seeking intentions; parents reported on family income, family function, mental health symptoms, and mental health stigma. Latent profile analysis and the BCH three-step method were used to identify subgroups and examine predictors and outcomes.

**Results:**

Five profiles were identified: normative, safe, distress, high-risk, and aware. The high-risk profile (5.98%) showed high distress, low help-seeking, and the highest self-harm rate (48.7%). Lower family functioning and higher parental distress predicted higher-risk profiles. Professional help-seeking intentions were associated with reduced self-harm risk among distressed adolescents.

**Conclusions:**

Family and parental factors significantly shape adolescent coping profiles and mental health risks. Findings underscore the value of early screening and family-focused interventions to reduce self-harm.

## Introduction

According to the World Health Organization [68], one in seven adolescents globally experience a mental health disorder, with depression, anxiety, and behavioral problems among the most prevalent conditions. Nearly 10% of adolescents engage in self-harm [21], and its incidence has continued to rise over the past decade across many regions, including China [9, 33], closely associated with anxiety and depression [63]. Contemporary research centers on early detection and intervention for these conditions to reduce psychological distress and prevent self-harm and suicide among adolescents [13, 14]. However, the effectiveness of such interventions depends not only on symptom severity but also on adolescents’ coping strategies.

Adolescents adopt both adaptive and maladaptive strategies to cope with mental health challenges. Adaptive coping includes help-seeking, emotion regulation, and problem-solving, whereas maladaptive coping involves avoidance, substance use, and self-harm [16, 58]. Help-seeking, an adaptive coping strategy, involves obtaining support from informal (family, friends) or formal (professionals) sources [1, 52]. Regrettably, those with the most pressing need for psychological support often exhibit the least inclination to pursue it [1]. Only a small minority of adolescents with emotional or behavioral problems—typically fewer than one-third—seek professional help [22, 64]. Up to half of adolescents who self-harm fail to seek help for their behavior [53]. Therefore, identifying at-risk youth and promoting help-seeking behaviors are essential to prevent further psychological harm.

Family environments play a vital role in adolescents’ psychological development and socialization, providing both material and emotional support [11, 15]. Extensive research indicates that family environments and parental characteristics can either promote or hinder adolescents’ mental health and help-seeking behaviors. Low socioeconomic status is associated with greater exposure to stress and reduced access to psychological help [49, 50]. Positive family functioning characterized by cohesion, flexibility, communication, and satisfaction has been identified as a key factor distinguishing adolescents with and without mental health problems [17], and such positive family environments, marked by harmony, low conflict, and strong parent-child interactions, have been shown to foster adolescents’ self-esteem and resilience [19, 57, 73]. Crucially, family support and parental availability enhance both the help-seeking intentions and subsequent actions of adolescents in need [20].

Parental mental illness and stigma have a significant impact on children’s psychological distress and help-seeking behavior. Offspring of parents with mental health treatment exhibit elevated levels of mental health symptoms [39], while parental mental illness has been linked to adolescent self-harm behavior [19]. Parents’ psychological distress may reduce their attention to their children’ s well-being and delay help-seeking on their behalf [2], whereas interventions addressing parental mental health yield positive outcomes for offspring [38, 39]. Furthermore, parental stigma toward mental illness impedes recognition and appropriate response to children’ s psychological problems [26, 65], diminishing adolescents’ likelihood of seeking professional support [42, 49]. A positive correlation between parent and child help-seeking intentions suggests that children often emulate their parents’ attitudes, meaning parental attitudes can either reinforce or undermine their own help-seeking behavior [4].

The person-oriented approach emphasizes patterns and processes within individuals, viewing them as integrated wholes rather than isolated variables [7]. While numerous studies have explored the influence of familial and parental factors on adolescents’ mental health and coping strategies using a variable-oriented approach (e.g., [4, 24, 30, 46, 77], the person-oriented approach has gained increasing traction in this domain. For example [78], identified three profiles of psychosocial adaptation among left-behind adolescents in rural China [29], discerned four profiles of adolescent males in Australian based on help-seeking patterns, and [3] found seven coping profiles among Japanese adolescents, revealing the persistence of maladaptive groups.

However, no existing research has holistically examined adolescents’ mental health distress, self-harm, and help-seeking together within a Chinese urban context. Previous studies have typically addressed these dimensions separately, overlooking how distinct constellations of distress and coping may influence self-harm risk. Adopting a person-oriented framework enables the identification of meaningful profiles that reflect the interplay of psychological distress and help-seeking intentions, offering new insights into the risk patterns underlying adolescent self-harm.

### Current study

Prior research has established the positive impact of supportive family environments in reducing psychological distress and promoting help-seeking intentions among adolescents. However, there remain gaps in the literature that warrant further holistic examination. To address these gaps, this exploratory person-oriented study aims to identify at-risk adolescents by concurrently investigating their mental health concerns and help-seeking intentions. Self-harm was included as a key outcome variable because it reflects severe psychological distress and serves as a well-established precursor of suicide risk [9]. Examining self-harm across identified profiles allows for a more applied understanding of how distinct constellations of distress and help-seeking interact to influence high-risk behaviors. This approach allows for a nuanced understanding of adolescents’ mental health profiles and provides evidence to inform more targeted prevention and intervention strategies.

Specifically, the study has two primary objectives: first, to identify the number and patterns of profiles based on adolescents’ psychological distress and help-seeking intentions; and second, to explore the predictors of profile membership and the prevalence of self-harm across profiles.

We hypothesized that:

1. Multiple profiles reflecting different combinations of distress and help-seeking intentions would emerge, including a group characterized by high distress and low help-seeking.
2. Family and parental variables would predict these different profiles.
3. Profiles displaying higher levels of distress and lower levels of help-seeking intentions would be associated with a higher incidence of self-harm.

## Methods

### Participants and procedure

The data used in this study is part of the Students**’** Mental Health Network (SMHN), a larger research project investigating Chinese adolescents’ family environments, mental health literacy, and psychological well-being. The study was conducted in Wuhan, a prominent city in central China, involving secondary school students (grades 7 through 12) and their parents. Participants were recruited from eight schools (four junior high schools and four senior high schools) across four districts. The sample included 7,934 pairs of adolescents (54.0% male, 46.0% female) and parents (43.5% male, 56.5% female). Both the adolescent and one parent completed questionnaires at the school. Written informed consent was obtained from both parents and adolescents prior to participation. Before beginning the survey, participants were familiarized with the study procedures. All participants completed an anonymous self-report questionnaire on an online assessment platform, taking an average of 20 min to finish. The adolescent survey contained 36 questions, and the parent survey contained 29 questions. As a token of appreciation, students and parents received popular science materials related to mental health. Data collection took place from September to October 2021. The research project was approved by the Institutional Review Board of Wuhan University School of Medicine.

## Measures

### Psychological distress

#### Depression (DP)

A Chinese version of the Patient Health Questionnaire 9-item (PHQ-9) [60, 27, 66] was used to assess the severity of depression. For each item, participants rated the frequency of their feelings (0 = never, 3 = nearly every day) such as anhedonia, depressed mood, and trouble sleeping over the past two weeks (e.g., “Little interest or pleasure in doing things.”). The total score, ranging from 0 to 27, was computed to represent the severity of depression. Both adolescents and their parents completed this questionnaire separately (α = 0.896 and 0.874, respectively).

#### Anxiety (AX)

A Chinese version of the Generalized Anxiety Disorder 7-item (GAD-7) [61, 62] was used to assess the severity of anxiety. For each item, participants rated the frequency of their feelings (0 = never, 3 = nearly every day) such as worries and trouble relaxing over the past two weeks (e.g., “Feeling nervous, anxious, or on edge.”). The total score, ranging from 0 to 21, was computed to represent the severity of anxiety. Both adolescents and their parents completed this questionnaire separately (α = 0.921 and 0.915, respectively).

#### Help-seeking intention (HSI)

A 10-item scale adapted from [69] was used to assess participants’ help-seeking intention. We specify the source of help to be more applicable to Chinese secondary students including “friend”, “parent”, “other relative/family member”, “classmate”, “headteacher”, “subject teacher”, “psychology teacher”, “psychiatrist”, “psychotherapist/therapist”, and “Internet/hotline”. Adolescents reported their willingness to seek non-professional help on six items and seek professional help on four items (0 = no, 1 = yes). The total score of each subcategory was computed to represent the non-professional help-seeking intention (NPHSI) and professional help-seeking intention (PHSI), respectively.

### Family environment

#### Family income (FI)

The annual household income was assessed by parents’ report. Participants were asked “In the most recent year, what was the range of your family’s total annual income” and choose one of the four options (income = less than 80,000 yuan, 80,000 to 150,000 yuan, 150,000 to 300,000 yuan, or 300,000 yuan and above).

#### Family function (FF)

Adolescents’ satisfaction with family functioning was measured by the Chinese version of the Family APGAR Index [41, 59] and reported by adolescents themselves. Participants rated the frequency of their feelings (0 = hardly ever, 2 = almost always) towards 5 domains (adaptation, partnership, growth, affection, and resolve) of family function (e.g., “I am satisfied with the help that I receive from my family when something is troubling me.”). Higher scores represent higher levels of family functioning. (α = 0.89).

#### Parent’s mental health stigma (PRMHS)

A Chinese version of the Perceived Devaluation Discrimination Scale (PDD) [31, 76] was used to measure parents’ mental health stigma. The scale comprises 12 items (e.g., “Most people would accept a person who once had a serious mental illness as a close friend”, “Most people believe that a person who has been in a psychiatric hospital is just as intelligent as the average person”). For each item, participants rated the extent to which they believe that most people will devalue or discriminate against someone with a mental illness (0 = strongly disagree, 4 = strongly agree). The average score was computed to represent their belief. (α = 0.841).

#### Self-harm

Adolescents’ self-harm behavior was assessed by their self-report. Participants were asked “In the past 12 months, have you intentionally injured yourself but not attempted to become suicidal?” and answered “yes” or “no” (0 = no, 1 = yes).

#### Covariates

Adolescents’ grade (18.6% Grade 7, 15.6% Grade 8, 13.7% Grade 9, 17.4% Grade 10, 17.7% Grade 11, 17.0% Grade 12), adolescents’ gender (54.0% male, 46.0% female), only child status (62.5% “yes”, 37.5% “no”) and single parent family status (23.5% “yes”, 76.5% “no”) comprised the covariates in the model.

#### Analytic plan

SPSS27 was used for preliminary descriptive analyses, while Mplus 8 [43] facilitated mixture model analysis. The BCH 3-step method was implemented to construct a mixture auxiliary model, initially identifying participant profiles before examining the associations among various predictors (e.g., family function), the outcome (self-harm), and the estimated profiles [6]. This technique accounts for misclassification and provides resistance to profile membership shifts throughout the model construction process [5]. Latent profile analysis (LPA) determined the number of profiles, employing maximum likelihood estimation with robust standard errors (MLR) and 500 random starts to avoid local maxima. During the model-building phase, latent profile models featuring different model specifications were scrutinized and compared [37]. The optimal fit model was selected based on model fit indices, such as the Lo-Mendell-Rubins Likelihood ratio test (LMR-LRT), the Bootstrap likelihood ratio test (BLRT), the Akaike Information Criteria (AIC), Bayesian Information Criteria (BIC), and the sample size-adjusted BIC (aBIC) [44]. Significant p-values for LMR-LRT and BLRT indicate a superior model fit for the k-class solution compared to the (k-1)-class solution. Additionally, entropy values were taken into consideration to enhance data-model fit identification. After selecting the best profile solution, predictors, covariates, and distal outcomes were included simultaneously in the analysis. The χ^2^ (Chi-square) test was also performed for pairwise comparison of self-harm as a distal outcome between the latent profiles.

## Results

### Descriptive statistics

Table 1 displays descriptive statistics, bivariate correlations, and self-harm occurrences. No missing values were detected. Adolescent distress and help-seeking variables showed significant correlations with each other, with a strong positive relationship between depression and anxiety (*r* = .81, *p* < .001). Similar patterns were observed in parents’ depression and anxiety levels, which correlated with their children’s distress variables (*r* = .20 and 0.18, *p* < .001). Most family and parental variables were significantly related to these factors, particularly family function (*r* = − .50, − 0.44, 0.49, and 0.31).

**Table 1:** Descriptive statistics, bivariate correlations, and self-harm occurrences

	M /%	SD	1	2	3	4	5	6	7	8	9
1 FI	2.30	0.95	–								
2 FF	1.41	0.55	**0.05** ^***^	–							
3 PRDP	1.81	3.04	**− 0.04** ^***^	**− 0.15** ^***^	–						
4 PRAX	2.40	3.19	0.00	**− 0.14** ^***^	**0.65** ^***^	–					
5 PRMHS	2.36	0.27	**− 0.04** ^***^	**− 0.04** ^***^	**0.04** ^***^	**0.04** ^***^	–				
6 ADDP	4.13	4.89	**0.05** ^***^	**− 0.50** ^***^	**0.20** ^***^	**0.18** ^***^	0.01	–			
7 ADAX	3.80	4.39	**0.07** ^***^	**− 0.44** ^***^	**0.19** ^***^	**0.19** ^***^	0.01	**0.81** ^***^	–		
8 ADNPHSI	3.16	1.87	**0.04** ^**^	**0.49** ^***^	**0.09** ^***^	**0.08** ^***^	**− 0.05** ^***^	**− 0.42** ^***^	**− 0.39** ^***^	–	
9 ADPHSI	1.32	1.55	**0.05** ^***^	**0.31** ^***^	**0.08** ^***^	**0.07** ^***^	**− 0.04** ^**^	**− 0.28** ^***^	**− 0.26** ^***^	**0.55** ^***^	–
*Self-harm behavior*											
No	0.90	–									
Yes	0.10	–									

*FI* Family income, *FF* Family function, *PRDP* Parent’s depression, *PRAX* Parent’s anxiety, *PRMHS* Parent’s mental health stigma, *ADDP* Adolescent’s depression, *ADAX* Adolescent’s anxiety, *ADNPHSI* Adolescent’s non-professional help-seeking intention, *ADPHSI* Adolescent’s professional help-seeking intention

Proportion (%) was applied for self-harm behavior in adolescents. Significant correlations (^**^p < .01, ^***^p < .001) were bolded

### Latent profiles of distress and help-seeking patterns

As presented in Table 2, the AIC, BIC, and aBIC favored a 6-profile solution, but a non-significant LMR-LRT indicated it did not significantly improve the fit compared to the 5-profile model (LMR-LRT *p* = .185). Moreover, the added profile in the 6-profile solution exhibited a pattern of medium to high psychological distress and medium help-seeking intention, which lacked a substantive interpretation that distinguished it from other profiles, and had an estimated prevalence of only 4.43%. Considering that profiles representing less than 5% of the sample were regarded as too small for meaningful interpretation, and an entropy value above 0.80 indicated good classification accuracy [45], a 5-profile solution was chosen as the best model. The final model (entropy = 0.896) demonstrated good information criteria (AIC = 136028.34, BIC = 136223.75, aBIC = 136134.77), and significant LMR-LRT (*p* < .001) and BLRT (*p* < .001).

**Table 2:** Model fit indices for the latent profiles of distress and help-seeking patterns

Profile	LL	AIC	BIC	aBIC	pLMR	pBLRT	Entropy
1	− 77766.18	155548.37	155604.20	155578.78	–	–	–
2	− 73573.99	147173.97	147264.70	147223.38	< 0.001	< 0.001	0.931
3	− 70354.40	140744.80	140870.42	140813.22	< 0.001	< 0.001	0.914
4	− 69010.45	138066.89	138227.41	138154.32	< 0.001	< 0.001	0.879
**5**	**− 67986.17**	**136028.34**	**136223.75**	**136134.77**	**< 0.001**	**< 0.001**	**0.896**
6	− 67264.23	134594.47	134824.77	134719.90	0.185	< 0.001	0.900

*LL* log-likelihood, *AIC* Akaike Information Criteria, *BIC* Bayesian Information Criterion, *aBIC* adjusted BIC, *pLMR p* values for Lo-Mendell-Rubin adjusted likelihood ratio test for K vs. K-1 profiles, *pBLRT p* values for Bootstrapped Likelihood-Ratio Test. The best model was bolded

**Fig. 1:**
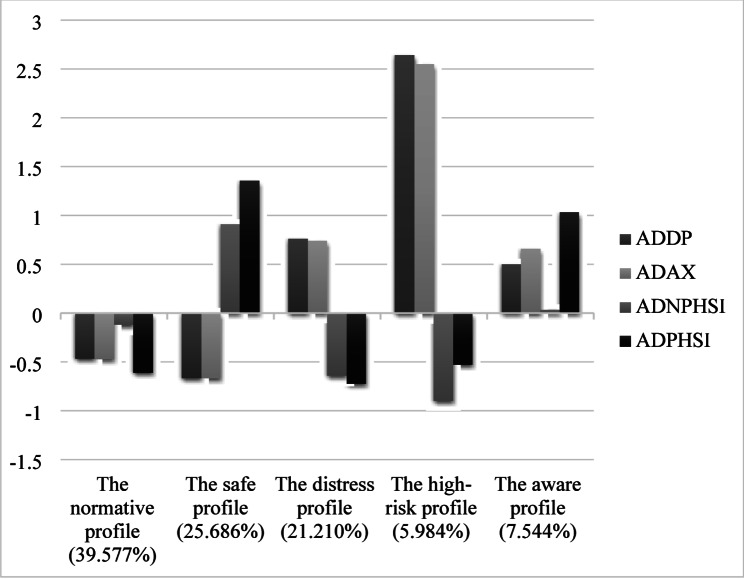
Standardized mean scores of the five profiles of distress and help-seeking patterns. *ADDP* Adolescent’s depression, *ADAX* Adolescent’s anxiety, *ADNPHSI* Adolescent’s non-professional help-seeking intention, *ADPHSI* Adolescent’s professional help-seeking intention

Based on the cut-off scores [28, 47], as shown in Fig. 1; Table 3, 39.577% of adolescents belonged to a normative group displaying a low level of psychological distress and a low to medium level of help-seeking intention. This profile was labeled as the normative profile. The second largest group (25.686%) exhibited low distress but high intention, and was therefore labeled as the safe profile. In contrast, the high-risk profile (5.984%) was characterized by a high level of distress and low help-seeking intention. The remaining two profiles, the aware profile (7.544%) and the distress profile (21.210%), were distinguished by their help-seeking intentions, with the former having significantly higher professional help-seeking intention than the latter. The estimated standardized differences in profile-specific indicator means for all pairwise comparisons across profiles demonstrated good class separation [37].

**Table 3:** The Z scores and confidence intervals of profile differences all indicator variables

	The normative profile (39.577%)		The safe profile (25.686%)		The distress profile (21.210%)		The high-risk profile (5.984%)		The aware profile (7.544%)	
	M	95%CI	M	95%CI	M	95%CI	M	95%CI	M	95%CI
ADDP	− 0.467	− 0.493, − 0.442	− 0.666	− 0.681, − 0.649	0.762	0.696, 0.835	2.641	2.490, 2.779	0.503	0.402, 0.606
ADAX	− 0.471	− 0.503, − 0.438	− 0.668	− 0.690, − 0.647	0.742	0.679, 0.809	2.549	2.418, 2.704	0.659	0.576, 0.752
ADNPHSI	− 0.121	− 0.155, − 0.088	0.912	0.870, 0.952	− 0.641	− 0.687, − 0.601	− 0.898	− 0.971, − 0.822	0.035	− 0.061, 0.143
ADPHSI	− 0.609	− 0.629, − 0.590	1.353	1.326, 1.377	− 0.724	− 0.739, − 0.707	− 0.528	− 0.589, − 0.457	1.033	0.964, 1.099

*ADDP* Adolescent’s depression, *ADAX* Adolescent’s anxiety, *ADNPHSI* Adolescent’s non-professional help-seeking intention, *ADPHSI* Adolescent’s professional help-seeking intention

### Predictions of distress and help-seeking patterns

As presented in Table 4, we found that family environment, parents’ psychological distress, and parents’ mental health stigma generally predicted profile membership effectively. Family environment and parents’ psychological distress were the strongest predictors across all profiles. Compared to the normative profile, adolescents from families with lower family functioning and with parents having higher level of anxiety and depression were more likely to display the distress profile, high-risk, and aware profile (Bs from − 0.83 to − 2.60, ORs from 0.07 to 0.44, *p* < .001 for family function; Bs from 0.04 to 0.07, ORs from 1.04 to 1.07, *p* < .05 for anxiety; Bs from 0.05 to 0.08, ORs from 1.06 to 1.08, *p* < .01 for depression). Adolescents from families with higher family income were more likely to display the distress profile and the high-risk profile than the normative profile (Bs from 0.19 to 0.34, ORs from 1.21 to 1.40, *p* < .001), while those with parents exhibiting weaker mental health stigma were more likely to display the distress profile than the normative profile (B = − 0.40, OR = 0.67, *p* < .01). When comparing the normative profile to the safe profile, adolescents from families with lower income, higher family functioning, and parents exhibiting weaker mental health stigma and lower level of depression were more likely to demonstrate the safe profile (B = − 0.19, OR = 0.83, *p* < .001 for family income; B = 1.57, OR = 4.80, *p* < .001 for family function; B = − 0.28, OR = 0.76, *p* < .05 for stigma; B = − 0.05, OR = 0.95, *p* < .01 for depression).

For covariates, in comparison to the normative profile, adolescents in higher grades were more likely to be in the other four groups, and girls were more likely to exhibit the distress, high-risk, or awareness profiles (Bs from 0.09 to 0.25, ORs from 1.10 to 1.28, *p* < .01 for grade; Bs from 0.56 to 0.84, ORs from 1.76 to 2.32, *p* < .001 for gender). Adolescents who were the only child were more likely to have the distress or awareness profiles over the safe profile (Bs = 0.22 and 0.35, ORs = 1.25 and 1.42, *p* < .05, respectively), and the aware profile over the normative profile (B = 0.28, OR = 1.32, *p* < .05). Those from single-parent families were more likely to possess the distress, high-risk, or awareness profiles compared to the safe profile (Bs = 0.31, 0.29 and 0.35, ORs = 1.37, 1.34 and 1.42, *p* < .05, respectively).

**Table 4:** Association between predictors and latent profile membership (all latent profiles are compared to the normative profile)

	The safe profile		The distress profile		The high-risk profile		The aware profile	
	B (SE)	OR	B (SE)	OR	B (SE)	OR	B (SE)	OR
FI	**− 0.19 (0.03)** ^***^	**0.83**	**0.19 (0.04)** ^***^	**1.21**	**0.34 (0.06)** ^***^	**1.40**	0.08 (0.06)	1.08
FF	**1.57 (0.09)** ^***^	**4.80**	**− 1.69 (0.08)** ^***^	**0.19**	**− 2.60 (0.12)** ^***^	**0.07**	**− 0.83 (0.10)** ^***^	**0.44**
PRDP	**− 0.05 (0.02)** ^**^	**0.95**	**0.06 (0.02)** ^**^	**1.06**	**0.08 (0.02)** ^***^	**1.08**	**0.05 (0.02)** ^**^	**1.06**
PRAX	− 0.01 (0.02)	0.99	**0.04 (0.02)** ^*^	**1.04**	**0.06 (0.02)** ^**^	**1.06**	**0.07 (1.07)** ^**^	**1.07**
PRMHS	**− 0.28 (0.12)** ^*^	**0.76**	**− 0.40 (0.14)** ^**^	**0.67**	− 0.18 (0.21)	0.83	− 0.30 (0.21)	0.74

*FI* Family income, *FF* Family function, *PRDP* Parent’s depression, *PRAX* Parent’s anxiety, *PRMHS* Parent’s mental health stigma

Significant correlations (^*^p < .05, ^**^p < .01, ^***^p < .001) were bolded

### Differences in self-harm behavior across profiles

As shown in Table 5, adolescents’ self-harm behavior differed across the five profiles. In particular, the high-risk profile was associated with higher prevalence of self-harm behavior over all other profiles (49.1%). The chi-square tests showed significant differences across all pairwise comparisons.

**Table 5:** Probabilities of self-harm behavior and chi-square tests across profiles

	Self-harm behavior		Vs. The normative profile	Vs. The safe profile	Vs. The distress profile	Vs. The high-risk profile	Vs. The aware profile
	No (%)	Yes (%)					
The normative profile	98.2	1.8	–	10.344^**^	236.058^***^	360.381^***^	41.580^***^
The safe profile	96.6	3.4		–	189.812^***^	340.267^***^	30.187^***^
The distress profile	80.5	19.5			–	124.577^***^	9.849^**^
The high-risk profile	50.9	49.1				–	139.202^***^
The aware profile	87.0	13.0					–

**p < .01, ***p < .001

## Discussion

Adolescents’ mental health and appropriate help-seeking behaviors are essential for healthy development. While the majority of prior research has focused on examining internal and external influences using a variable-oriented approach, this study explored adolescents’ mental health and its correlates from an exploratory person-oriented perspective. Our goal was to identify which adolescents are most at risk and to clarify how family and parental factors contribute to these patterns. In accordance with previous research (e.g., [15, 71]), our findings revealed associations between adolescents’ mental health, parental symptoms, and perceived family support, highlighting the close interdependence between family well-being and adolescents’ psychological adjustment.

Resonating with recent research findings (e.g., [18, 70]), the profiles identified in this study underscored the mental health crisis among Chinese adolescents, particularly the large proportion who experience distress but are unwilling to seek help. Overall, three of the five profiles, representing 66.77% of adolescents, demonstrated limited help-seeking intentions, revealing a persistent gap between psychological need and service utilization. This finding aligns with previous research indicating that up to half of adolescents who engage in self-harm do not seek professional assistance [53], emphasizing the necessity of enhancing mental health literacy and early intervention within schools and communities.

Concerningly, two profiles exhibited high levels of psychological distress. The high-risk profile (5.98%) reported severe psychological distress, frequent self-harm, low perceived family support, and parents with elevated symptom levels. This subgroup should be prioritized for clinical attention, with coordinated efforts involving parents and other key adults, such as teachers, to ensure timely access to professional help and early identification of self-harming behaviors. The distress profile (21.21%) exhibited elevated but largely subclinical symptoms, lower perceived family support, and parents showing mild psychological distress. Preventive interventions targeting this group should emphasize peer and family psychoeducation to strengthen support networks, enhance help-seeking motivation, and reduce the likelihood of symptom escalation.

In contrast, the safe (25.69%) and normative (39.58%) profiles were characterized by relatively stable mental health and lower distress, while the aware profile (7.54%) combined moderate distress with stronger readiness to seek help. Adolescents in the safe profile appeared more open to seeking assistance even in the absence of distress, while those in the aware profile showed greater self-awareness and willingness to seek external support when facing difficulties. Some adolescents may avoid turning to friends or parents due to fears of being perceived as attention-seeking [53], instead preferring professional services [40]. However, no subgroup characterized by high professional but low non-professional help-seeking intentions was identified.

The examination of profile predictors demonstrated that a better family environment and parents with fewer mental health issues and positive attitudes toward mental health were closely associated with adolescents’ psychological well-being and more effective coping strategies. The importance of family context was further reinforced by the finding that higher family functioning was linked to safer and more adaptive profiles. Interestingly, higher family income increased the likelihood of being classified as at-risk, suggesting that material advantages alone do not ensure emotional resilience. Although higher-income families might have greater access to help-seeking resources, previous studies have reported both positive and negative relationships (e.g., [12, 23, 36]). Similarly, findings in China remain inconsistent (e.g., [55, 74]. Since most existing studies focus on adults, our findings contribute to the current understanding of how family material conditions and emotional support influence adolescents’ psychological well-being.

Logan and King [32] model of adolescent help-seeking emphasized the value of parental involvement throughout the entire help-seeking process. Compared to adults, adolescents with mental health problems are more vulnerable, which increases their need for parental facilitation in seeking necessary professional assistance [48, 51]. This is particularly relevant in many jurisdictions where adolescents are legally required to obtain parental permission to access medical treatment [42]. However, it is also worth noting that a recent systematic review reported that excessive parental involvement in the help-seeking process and neglect of young people’s self-determination might make them less likely to seek help [49]. The role of family and parents and the underlying mechanisms of their function in protecting adolescents warrant further investigation.

Our results also indicated that parents’ psychological distress predicted profiles with lower help-seeking intentions. As supported by prior work, this may be explained both biologically (e.g., [8]) and environmentally (e.g., [10, 54]). In addition, surprisingly, we found that parents’ weaker mental health stigma was associated with both the safe profile and the distress profile, which may seem counterintuitive for the latter. This finding may imply that parents’ attitudes towards mental health might be passed on to adolescents through example, subtly influencing them in their day-to-day family life. One possible explanation for this association with the distress profile could be the non-disclosure of adolescents’ self-reported psychological distress due to stigma. Alternatively, considering previous research highlighting the impact of parenting styles on adolescents’ mental health status and coping [35, 72], parents’ mental health stigma might shape children’ s attitudes through authoritarian parenting, leading children to conform and avoid going against their parents’ wishes.

In line with recent prior work, the overall prevalence of adolescent’ self-harm behaviour reported in our study was at a considerable level [34]. The high-risk profile had the highest rate (48.7%), consistent with [67] suggesting that adolescents with severe distress who avoid help-seeking are more prone to self-harm and suicidality. The distress profile showed a moderate rate (19.0%), higher than the aware profile (13.8%), given that professional help-seeking intention was the distinguishing indicator between the two profiles. Previous research has suggested that low rates of access to non-professional sources of help were associated with an increased likelihood of self-harming tendencies [56]. However, our findings expand upon this knowledge and further emphasize the harm prevention role of openness toward mental assistance from professional sources.

### Limitations and implications

While this study boasts several notable strengths, such as a large sample size with an evenly distributed sample across gender and grades to better represent the secondary school adolescent population, a few limitations should be acknowledged. Firstly, the cross-sectional design limits our ability to investigate causal relationships, which could be addressed through longitudinal data. Secondly, the measurement of parents’ perspectives was based solely on the report of one parent, and future research could benefit from incorporating self-reports from one or both parents, depending on the family structure, to minimize bias. Thirdly, the study sample consisted solely of Chinese adolescents, primarily from urban areas, which restricts the generalizability of the findings beyond this cultural and demographic context. Future research should expand the sample and explore the applicability of this model to other groups (e.g., those from rural areas), considering the significant urban-rural gap in China regarding parental involvement and family patterns [25, 71]. Finally, the study was conducted during a period of partial COVID-19 restrictions, which may have influenced adolescents’ mental health and help-seeking behaviors. The impact of these restrictions was not specifically measured, and future research should consider the effects of pandemic-related stress and isolation on adolescent mental health.

The current study makes valuable methodological and substantive contributions to the literature on adolescents’ mental health. Firstly, our findings emphasize the need to enhance psychoeducation. Given that shame, stigma, and devaluation associated with mental health problems and treatment persist among a considerable population, impeding public mental health efforts—especially in the Chinese context [75]—adolescents would benefit from improved psychoeducation on not only themselves but also their parents. Secondly, as the first study to comprehensively examine the impact of family and parental factors on adolescents’ mental health issues and help-seeking intention, our research paves the way for future person-oriented approaches to studying adolescent mental health crisis. Subsequent applications could focus on identifying high-risk adolescents and implementing targeted interventions. Furthermore, this study enriches our understanding of the risk factors affecting adolescents’ healthy development from an interpersonal perspective. Future research should delve deeper into the role of psychopathology within families and parents to better protect adolescents from mental health crisis. Prevention and intervention programs could place a greater emphasis on addressing the family unit as a whole.

## Data Availability

The datasets generated and/or analyzed during the current study are not publicly available but are available from the corresponding author on reasonable request.
